# Analysis of Precipitation Control Process and Mechanical Properties of Ti-2Al-9.2Mo-2Fe Alloy

**DOI:** 10.3390/ma18112448

**Published:** 2025-05-23

**Authors:** Su-Hong Shin, Dong-Geun Lee

**Affiliations:** Department of Materials Science and Metallurgical Engineering, Sunchon National University, Sunchon 57922, Republic of Korea; tnghd1999@naver.com

**Keywords:** Ti-2Al-9.2Mo-2Fe, aging, ω phase, phase transformation, secondary α phase

## Abstract

Ti-2Al-9.2Mo-2Fe (2A2F) alloy is a low-cost β-Ti alloy in which the expensive β-stabilizing elements (Ta, Nb, W, Ni) are replaced with relatively inexpensive Mo and Fe for use in low-cost applications in various industries. The 2A2F alloy exhibits excellent mechanical properties such as high specific strength and low elastic modulus compared to conventional steel alloys but is prone to brittleness owing to the formation of the ω phase when heat-treated at relatively low temperatures. Therefore, an appropriate aging treatment should be performed to control the precipitation of the isothermal ω phase and secondary α phase. This study aims to derive the appropriate aging-treatment conditions following a solution treatment at 790 °C for 1 h, which is below the β-transus temperature of 815 °C. The aging treatments are conducted at holding temperatures in the range of 450–600 °C and holding times between 1 and 18 h. At relatively low aging temperatures of 450 °C and 500 °C, the precipitation of the isothermal ω phase resulted in significantly high hardness and compressive strength. As the aging temperature and holding time increased, the ω phase gradually transformed into the secondary α phase, leading to a balanced combination of strength and ductility. However, at excessively high aging temperatures and prolonged durations, excessive precipitation and growth of secondary α phases occurred, which caused a reduction in hardness and compressive strength, accompanied by an increase in ductility. In this study, the effects of precipitation evolution on mechanical properties such as tensile strength and hardness under various heat treatment conditions were comparatively analyzed.

## 1. Introduction

Carbon neutrality has now become a key global issue, and various industries are aiming to reduce carbon emissions for reasons such as energy conservation and stricter environmental regulations [1,2,3] because of global warming. This trend is also manifested in the automotive industry, where research on lightweight vehicle parts that can meet various requirements, such as reduced greenhouse gas emissions and improved fuel efficiency, is continuously being conducted in anticipation of more stringent regulations [1,2,3,4,5]. Accordingly, various materials have been tested to reduce the weight of automotive components. Recently, Ti alloys with excellent mechanical properties such as high specific strength, excellent corrosion resistance, and low elastic modulus have been actively researched and developed [6,7,8,9].

Ti alloys are classified into α alloys, α + β alloys, and β alloys, based on the Al and Mo added by the stabilizing elements. β-Ti alloys containing a large amount of β-stabilizing elements (Ta, Nb, W, Ni, etc.) have excellent machinability compared to other alloys, and their mechanical properties can be controlled in various ways by adjusting machining and heat-treatment conditions [10,11,12]. Additionally, β-Ti alloys used in automotive springs have a density of approximately 56% and a shear modulus of approximately 50% of those of steel. This allows significant weight reduction by reducing the number of springs and ride height. Coil springs made from various β-Ti alloys, such as Ti-3Al-8V-6Cr-4Mo-4Zr (Beta-C) alloys, have high yield strengths of up to 1350 MPa and a low elastic modulus of approximately 100 GPa, resulting in a weight reduction of approximately 47% compared to existing steel alloys [13]. Although Cr-Si steel exhibits high strength, β-Ti alloys provide a significantly higher strength-to-weight ratio, making them more suitable for applications where weight savings and flexibility are critical [9,13,14].

However, the manufacture of β-Ti alloys requires significant amounts of β-stabilizing elements (Zr, V, Nb, Ta, etc.), which have high unit costs, making them unsuitable for use in the automotive industry, where low cost is a criterion [15,16]. Low-cost β (LCB) Ti alloys, using elements such as Mo, Cr, and Fe instead of expensive β-stabilizing elements, have been developed to solve this problem [16,17,18,19,20]. As LCB-Ti alloys exist in the β phase at room temperature, excellent mechanical properties can be obtained by precipitating the α phase with a hexagonal close-packed structure within the β-phase matrix with a body-centered cubic structure through solubilization and aging treatments and by controlling its morphology, distribution, and fraction [21,22]. In this way, LCB Ti alloys offer a cost advantage over other β-Ti alloys by utilizing affordable alloying elements and achieving desirable mechanical properties through heat treatment.

The Ti-2Al-9.2Mo-2Fe (2A2F) alloy used in this study is an LCB-Ti alloy developed for automotive spring applications. It achieves high strength through the addition of economical β-stabilizing elements (Mo and Fe) and a minor Al addition to further enhance the mechanical properties [23]. Previous studies on the 2A2F alloy have primarily focused on processing conditions, microstructures after solution treatment, and tensile properties [24,25,26]. However, the effects of aging temperature and time on the compressive properties and associated microstructural changes have not been sufficiently investigated. The 2A2F alloy has a low Mo equivalent owing to the low content of β-stabilizing elements. Therefore, an isothermal ω phase can be easily precipitated within the β-phase matrix upon aging at low temperatures (300–500 °C) [26,27]. The precipitation of the ω phase generally enhances hardness and strength, but significantly reduces ductility and toughness, leading to brittleness. However, when appropriately controlled through aging, the ω phase can be used to improve the overall mechanical performance of the alloy [28,29].

Therefore, in this study, we aimed to derive optimal heat-treatment conditions to control the mechanical properties of the 2A2F alloy, which can be applied to materials for transportation devices such as automobile springs. The microstructural changes such as the distribution, size, and phase-volume fraction of various precipitates (α phase and ω phase) were analyzed by solution treatment at a temperature below the β-transformation temperature, followed by aging treatments at various temperatures and times. In addition, the effects of microstructural changes on the mechanical properties were compared and analyzed to obtain excellent mechanical properties.

## 2. Experimental

The Ti-2Al-9.2Mo-2Fe alloy used in the experiments was melted by vacuum arc remelting, and the ingot was forged into a square billet with dimensions of 400 × 400 × L mm at approximately 1100 °C. The square billet was then hot rolled at 800 °C to form a round bar with a diameter of Ø 12 mm. The chemical composition of the alloy is listed in Table 1 and was analyzed by ICP-OES (iCAP PRO, Thermo Fisher Scientific, Waltham, MA, USA).

The β-transformation temperature was estimated to be approximately 815 °C (±10 °C) using the empirical model described in Equation (1) [30]. Although Ref. [31] does not present an explicit formula, it supports the compositional trends on which the model is based.

(1) $$T_{\beta }=872+23.4\left[Al\right]+32.1\left[Si\right]-7.7\left[Mo\right]-12.4\left[V\right]-14.3\left[Cr\right]-8.4\left[Fe\right]$$

**Figure 1:**
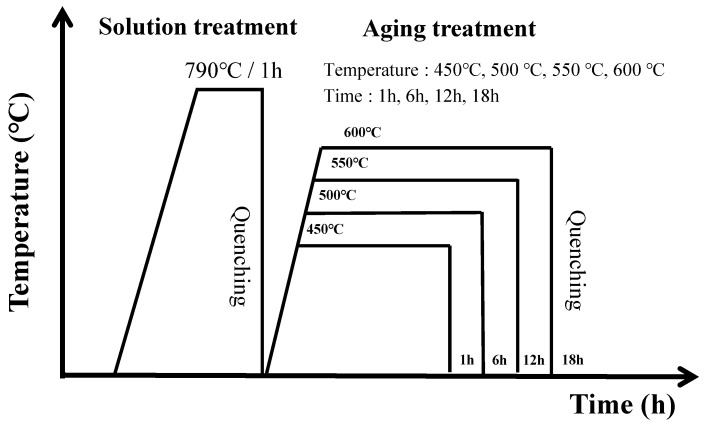
Schematic heat treatment process of Ti-2Al-9.2Mo-2Fe alloy.

Subsequently, the rolled 2A2F alloy specimens were solution-treated at 790 °C for 1 h below the β-transformation temperature (815 °C) and then quenched. Aging was then performed at four different temperatures (450, 500, 550, and 600 °C) for four different holding times (1, 6, 12, and 18 h). In total, 16 different conditions were used in the aging treatment. The detailed subsequent heat-treatment process is schematized in Figure 1. The specimens were then micro-polished using #220 to #4000 abrasive paper and 6, 3, 1, and 0.04 μm abrasives, for microstructure observation. The resulting specimens were etched with Kroll’s solution (5 mL HF + 15 mL HNO_3_ + 80 mL H_2_O) for approximately 5–25 s, and examined using an optical microscope (BX52M, Olympus, Tokyo, Japan) and a field-emission scanning electron microscope (JSM-7001F, JEOL, Tokyo, Japan) to observe the changes in the grain structure and boundaries, as well as to analyze the interface characteristics, fine distribution, and morphology of the precipitated phases. X-ray diffraction (D8 Discover, Bruker AXS, Karlsruhe, Germany) was performed to observe the changes in the precipitated phases under different aging conditions. The test conditions for XRD were 2θ = 30–90°, with a step size of 0.02° and a time per step of 2.4 s, using Cu-Kα radiation at 40 kV and 30 mA.

To evaluate the mechanical properties, Vickers hardness and room-temperature compression tests were performed. Vickers hardness measurements were performed using a Vickers hardness tester (HM-200, Mitutoyo, Japan) with a test load of 1 kgf. The hardness was measured at 14 locations, and the average of 12 values was calculated, excluding the maximum and minimum values. The compressive properties of each specimen were determined using a Dynamic Universal Materials Testing Machine (BESTUTM-10MD, Ssaul Bestech, Hwaseong, Republic of Korea). The compression specimens comprised Ø6 × 9 mm specimens, according to ASTM-E9-09 [32], and the average values of compressive strength and compressive elongation were calculated by performing three room-temperature compression tests at a strain rate of 1 ×10^−3^ mm/s. All contact surfaces were lubricated to reduce the friction between the die and both ends to prevent barreling.

## 3. Results and Discussion

### 3.1. Microstructures According to Solution Treatment

**Figure 2:**
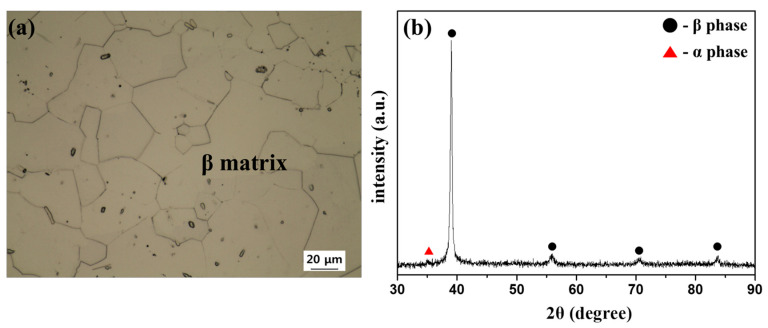
(**a**) Optical microstructure and (**b**) XRD diffraction profile of Ti-2Al-9.2Mo-2Fe alloy after solution treatment at 790 °C for 1 h followed by quenching.

**Figure 3:**
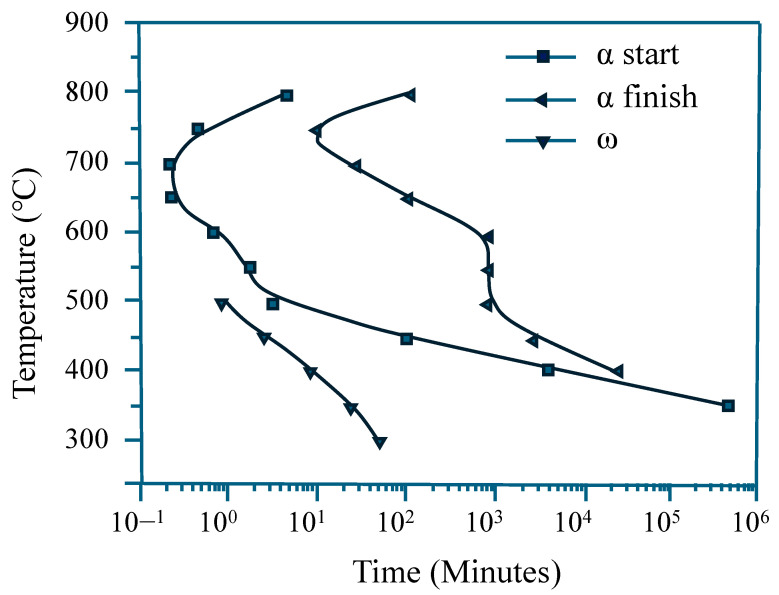
TTT diagram representing β decomposition and phase precipitation kinetics of α and ω phase in the Ti-2Al-9.2Mo-2Fe alloy [36].

Figure 2 presents the microstructure and XRD analysis results of the 2A2F alloy after quenching following a 1 h solution treatment at 790 °C, which is below the β-transformation temperature (815 °C) calculated using an empirical temperature formula. XRD analysis revealed only the characteristic peaks corresponding to the α and β phases [33]. The α phase consists mainly of Ti and Al atoms, whereas the β phase is stabilized by the presence of Mo and Fe. Although an athermal ω phase typically forms via diffusionless transformation during rapid cooling from above the β-transformation temperature, no such phase was observed in this study, as the solution treatment was conducted below that temperature [34,35]. Microstructural observation revealed fine phases in a homogeneous equiaxed β matrix (average grain size: 35 μm), which were identified as primary α precipitates based on the TTT diagram in Figure 3 and supporting XRD results. The diagram, adapted from Ref. [36], was originally constructed for the Ti-2Al-9.2Mo-2Fe alloy and reliably predicts α-phase formation during isothermal aging. These primary α phases acted as pinning points and reduced the mobility of the β-phase grain boundaries, limiting the recrystallization and grain growth of the β phase [37].

### 3.2. Changes in Precipitation Phases Due to Aging Treatment

**Figure 4:**
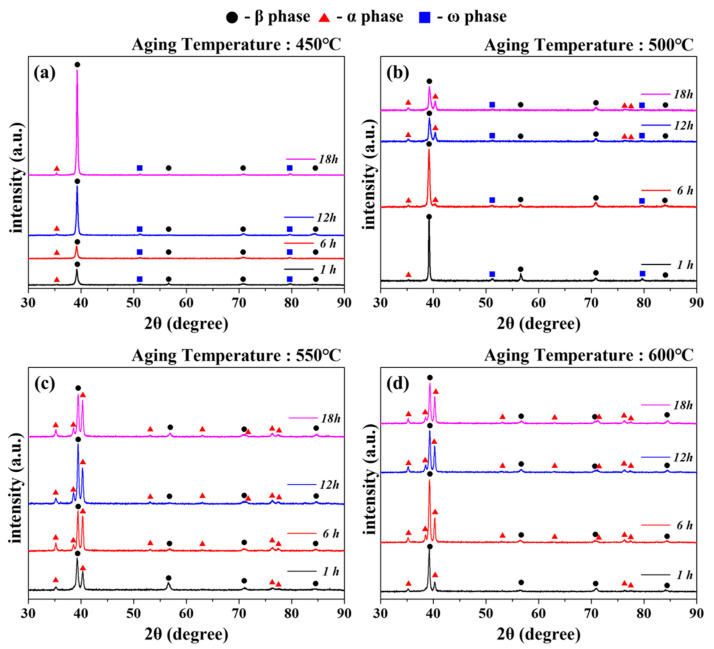
XRD diffraction profiles of Ti-2Al-9.2Mo-2Fe alloy after solution treatment at 790 °C for 1 h and aging at (**a**) 450 °C, (**b**) 500 °C, (**c**) 550 °C, and (**d**) 600 °C for 1 h~18 h.

**Figure 5:**
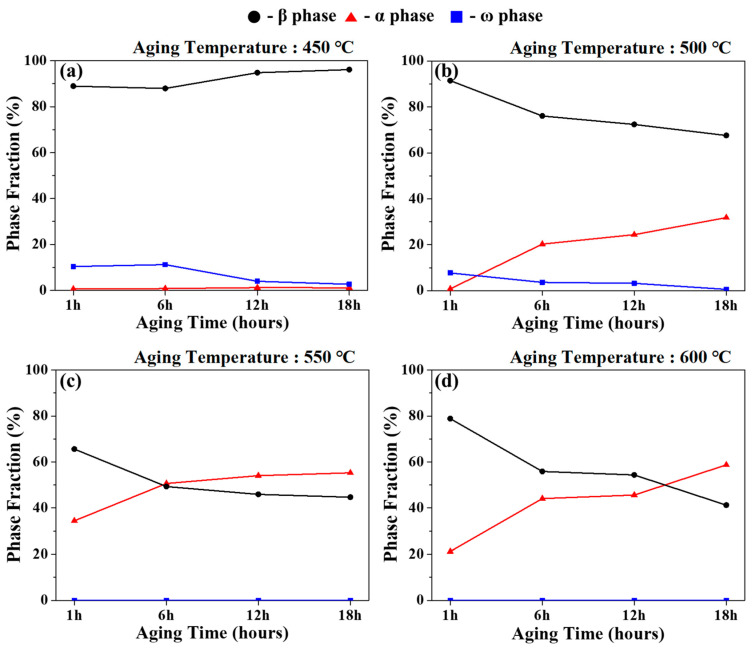
Phase fraction of Ti-2Al-9.2Mo-2Fe alloy after solution treatment at 790 °C for 1 h and aging at (**a**) 450 °C, (**b**) 500 °C, (**c**) 550 °C, and (**d**) 600 °C for 1 h–18 h.

The XRD measurement results of the specimens and changes in the volume fraction of each phase, according to the solution-treatment followed by the aging treatment are presented in Figure 4 and Figure 5, respectively. To quantify the changes in the volume fraction of each phase, the changes according to the aging conditions were calculated using Equation (2) and are presented in Table 2.

(2) $$V_{f,\alpha }=\frac{A_{\alpha }}{A_{\alpha }+A_{\beta }+A_{\omega }},\;V_{f,\beta }=\frac{A_{\beta }}{A_{\alpha }+A_{\beta }+A_{\omega }}\;,\;V_{f,\omega }=\frac{A_{\omega }}{A_{\alpha }+A_{\beta }+A_{\omega }}$$

where *V_f,α_*, *V_f,β_*, and *V_f,ω_* are the volume fractions of α, β, and ω phases, respectively, and A_α_, A_β_, and A_ω_ represent the integrated peak areas of each phase obtained from the XRD patterns.

β-phase and α-phase peaks were commonly observed in all aged specimens, with an additional ω-phase peak observed for the 450 and 500 °C aging conditions. This was the isothermal ω phase formed during aging at low temperatures, which is expected to significantly improve hardness and yield strength depending on the amount of precipitation but will be accompanied by a decrease in elongation [38,39]. As shown in Figure 4 and Figure 5a, for the 450 °C aging condition, the precipitation of the ω phase started at 1 h, followed by a maximum ω phase volume fraction (11.3%) at 6 h, which then decreased with increasing aging time. On the other hand, for the 500 °C aging condition, presented in Figure 4 and Figure 5b, the highest ω phase fraction was observed at 1 h, and the ω-phase fraction gradually decreased with the increase in aging time. This decrease in the ω-phase fraction can be noted in Table 2. Both the 450 and 500 °C aging conditions were accompanied by a decrease in the ω-phase fraction and an increase in the α-phase fraction.

This was because the *β*/*ω* phase boundary acted as a preferential nucleation site for the secondary α phase, which was formed through a phase transformation. The concentration gradient of β-stabilizing elements (Mo and Fe) between the β and ω phases promoted the diffusion of solute atoms, thereby facilitating the precipitation of the secondary α phase. The concentration-gradient difference between the β-stabilizing element-rich β-phase matrix and β-stabilizing element-poor ω phase caused the diffusion of solute atoms during aging, resulting in a redistribution at the *β*/*ω* boundary and precipitation of the secondary α phase. Chemically, the α phase is Al-enriched and Ti-based, the β phase is rich in β-stabilizing elements such as Mo and Fe, and the ω phase contains a relatively lower amount of these elements. This compositional difference induces diffusion during aging and contributes to the transformation behavior observed. A subsequent increase in the aging temperature and time led to long-range diffusion of solute atoms, which promoted the precipitation and growth of the secondary α phase [28,29].

**Figure 6:**
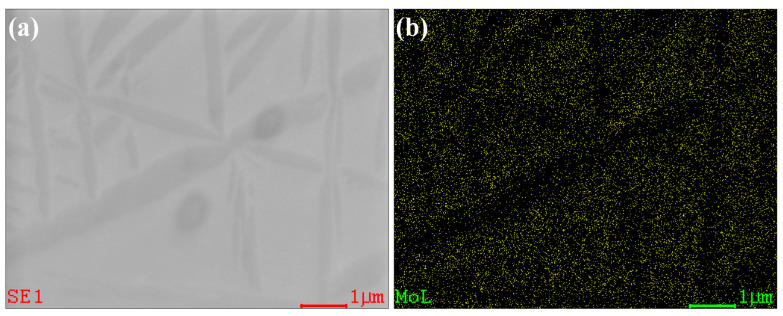
(**a**) SEM image of the Ti-2Al-9.2Mo-2Fe (2A2F) alloy; (**b**) EDS mapping of Mo showing its elemental distribution.

**Figure 7:**
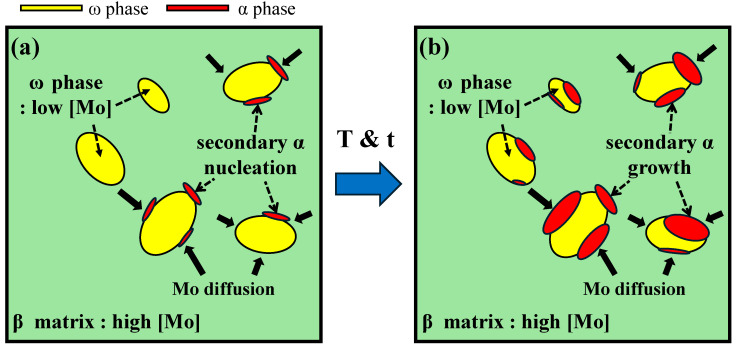
Schematic diagram illustrating (**a**) the nucleation of secondary α and (**b**) the growth of secondary α precipitate during aging treatment.

To provide microstructural evidence supporting this mechanism, SEM-based EDS mapping was performed, focusing on the distribution of Mo as a representative β-stabilizing element. As shown in Figure 6, the Mo concentration is significantly lower in the secondary α phase compared to the surrounding β matrix. This distinct elemental distribution confirms the existence of a Mo concentration gradient, which likely drives diffusion toward the β phase and promotes the nucleation of secondary α at the *β*/*ω* interface. The detailed phase transformation pathway, illustrating the role of the ω phase in the nucleation of secondary α, is schematically presented in Figure 7.

However, although phase transformation to the secondary α phase occurs at an aging temperature of 450 °C, the α phase-fraction change is very small, ranging from 0.6% to 1.2%. This is due to the lack of driving force for phase transformation at the low aging temperature of 450 °C. For the aging conditions of 550 and 600 °C, no ω-phase peak is observed, as shown in Figure 4 and Figure 5c and Figure 4 and Figure 5d, respectively. Instead, several secondary α-phase peaks derived from the transformation of the ω phase are detected, consistent with the transformation mechanism illustrated in Figure 7. This suggests that the high aging temperature caused all the ω phases to phase transform into the secondary α phase before the aging time of 1 h. The high α-phase fraction, as shown in Table 2 and Figure 5, suggests that the increase in aging temperature promoted the precipitation and growth of the α phase.

The α-phase fraction increased more significantly with aging temperature (by 41.6%) than with aging time (22.2%), indicating that temperature had a stronger effect on phase transformation, with a difference of approximately 19.4%. This confirmed that although an increase in aging time influenced the α-phase precipitation and growth, the effect of aging temperature for 6 h was more dominant in determining the microstructural evolution. This phase transformation and fractional change with aging conditions could affect the mechanical properties, and to further analyze this, the microstructure was observed by optical microscopy.

**Figure 8:**
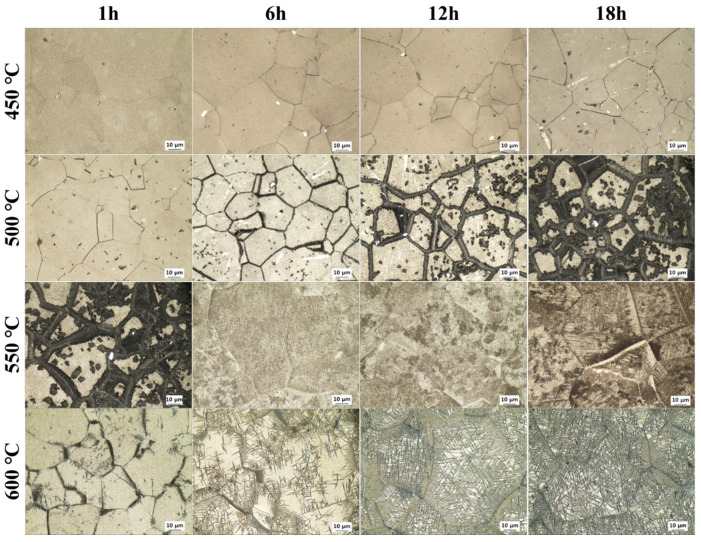
OM micrographs of Ti-2Al-9.2Mo-2Fe alloy after solution treatment at 790 °C for 1 h and aging holding times and temperatures.

To analyze the microstructure changes as a function of aging conditions, optical microscopy was performed, and the results are presented in Figure 8. Based on the overall equiaxed β-phase matrix, it can be observed that the secondary α phase precipitates and grows with increasing aging temperature and time. For the lowest aging temperature of 450 °C, the fraction of α and ω phases was so small that it was not observed under the optical microscope; only the equiaxed β-phase matrix was observed. The reason for this lack of microstructural change was the insufficient driving force for phase transformation and growth due to the low aging temperature, as mentioned earlier. For an aging temperature of 500 °C, indiscriminate precipitation of the secondary α phase within the β-phase matrix and in the grain boundaries began at an aging time of 1 h. Consistent with the XRD analysis results, the amount of secondary α-phase precipitation increased with increasing aging time. Furthermore, it was observed that precipitation preferentially occurred within the grain boundaries. This was because defects that act as nucleation sites exist not only at the *ω*/*β* phase boundary but also at the grain boundary, providing nucleation sites favorable for secondary α phases [40,41,42]. For high aging temperatures of 550 and 600 °C, it was observed that the ω phase transformed into the secondary α phase and covered most of the β-phase matrix before the aging time of 1 h, because of the high precipitation driving force. In particular, the microstructures at 500 °C/18 h and 550 °C/1 h exhibited similar morphologies. Combined with the XRD results, this confirmed that aging temperature had a more pronounced effect on the precipitation and growth of the secondary α phase than aging time.

**Figure 9:**
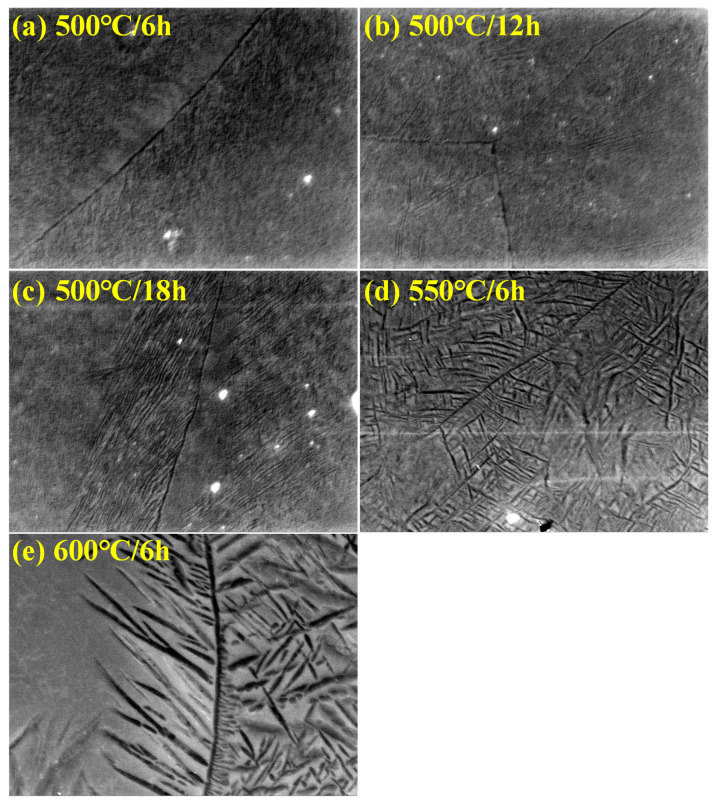
SEM micrographs of Ti-2Al-9.2Mo-2Fe alloy after solution treatment at 790 °C for 1 h and aging at (**a**) 500 °C for 6 h, (**b**) 500 °C for 12 h, (**c**) 500 °C for 18 h, (**d**) 550 °C for 6 h, and (**e**) 600 °C for 6 h (all at ×8000 magnification).

To analyze the microstructural changes more closely, the micrographs observed by scanning electron microscopy (SEM) are presented in Figure 9. At the aging temperature of 500 °C, it was observed that the Widmanstatten grain boundary α (WGB α) was formed along the grain boundary, which grew in the direction of the β-phase matrix. Although α-phase precipitation occurred at low aging temperatures, the insufficient driving force for growth limited the development of large α precipitates. However, as the aging temperature increased, the α phases nucleated and grew around the grain boundaries by diffusing the β stabilizing elements near the grain boundaries and reducing their stability, forming the WGB α phase [40,41,42,43]. For high aging temperatures of 550 and 600 °C, the growth of the α phase was accelerated because of the high driving force, forming a basket-weave α phase in the grain boundary in addition to the WGB α phase. It is expected that the secondary α phase will be distributed homogeneously throughout most of the β-phase matrix as the aging time increases, which will have a significant impact on the mechanical properties. The general trend observed in OM and XRD results indicates an increase in the α-phase fraction with aging temperature and time; however, a decrease was observed beyond 550 °C. The reduction in α precipitates at 600 °C is attributed to the lower degree of supercooling at high temperatures, which delays the nucleation of secondary α phases despite the increased growth space [44]. In SEM images, the number of fine α precipitates appears reduced, but this is due to the formation of coarser α structures, such as WGB α, resulting from grain boundary stabilization [45]. Therefore, the difference in observations arises not from a contradiction between the methods, but from changes in morphology and scale.

### 3.3. Hardness Properties

**Figure 10:**
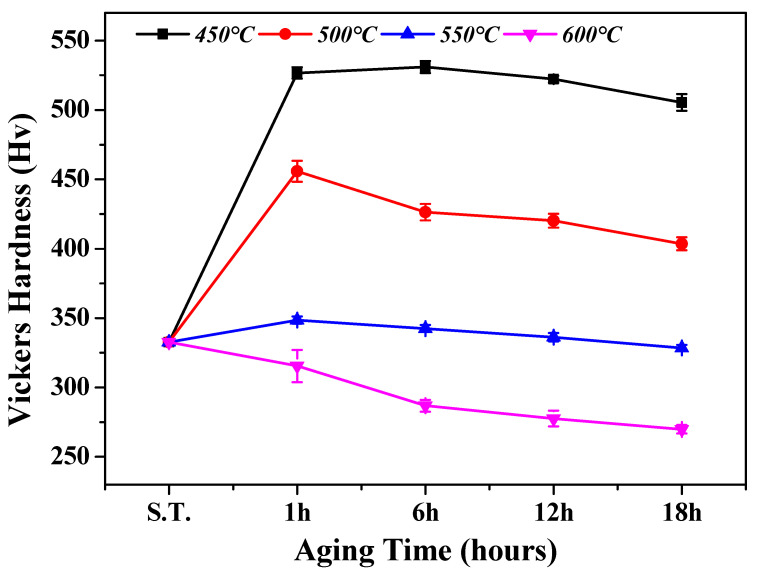
Vickers hardness variation of Ti-2Al-9.2Mo-2Fe alloy after solution treatment at 790 °C for 1 h and aging holding times and temperatures.

The hardness evolution under different aging-treatment conditions is presented in Figure 10 to analyze the influence of microstructure changes on the mechanical properties. For the relatively low aging temperatures of 450 and 500 °C, the samples exhibited high hardness values, averaging about 500 HV, because of the formation of the isothermal ω phase, which significantly enhanced the hardness. In particular, the 450 °C/6 h aging condition exhibited the maximum hardness value (530 HV) due to the strengthening effect of the high ω-phase volume fraction (11.3%). However, with an increase in aging time, the hardness value tended to decrease gradually owing to the growth of the fine ω phase and phase transformation into the secondary α phase. In addition, for the 500 °C condition, a large decrease in the hardness value (29.5 HV) was observed when the aging time increased from 1 to 6 h. This was due to the significant phase transformation of the ω phase to the secondary α phase between the 1 h and 6 h aging conditions.

On the contrary, relatively higher aging temperatures of 550 and 600 °C produced average hardness values of approximately 330 HV and 280 HV, respectively, which were similar or lower than that of the solution-treated specimen (330 HV). This was because the high aging temperature caused all of the precipitated ω phase to transform into a relatively softer secondary α phase before the aging time reached 1 h, resulting in softening. In addition, as the aging time increased, the secondary α phase grew to cover most of the β-phase matrix uniformly, reducing the strengthening effect. Although the 550 °C condition had a higher α-phase fraction than the 600 °C aging condition, it produced a higher hardness value, which is believed to be due to the higher α-phase precipitation amount increasing the grain boundary, resulting in a higher strengthening effect compared to that of the α-phase growth. Therefore, the changes in the overall hardness values were influenced more by the aging temperature than the aging time, similar to the microstructural property changes described earlier.

### 3.4. Room-Temperature Compression Properties

**Figure 11:**
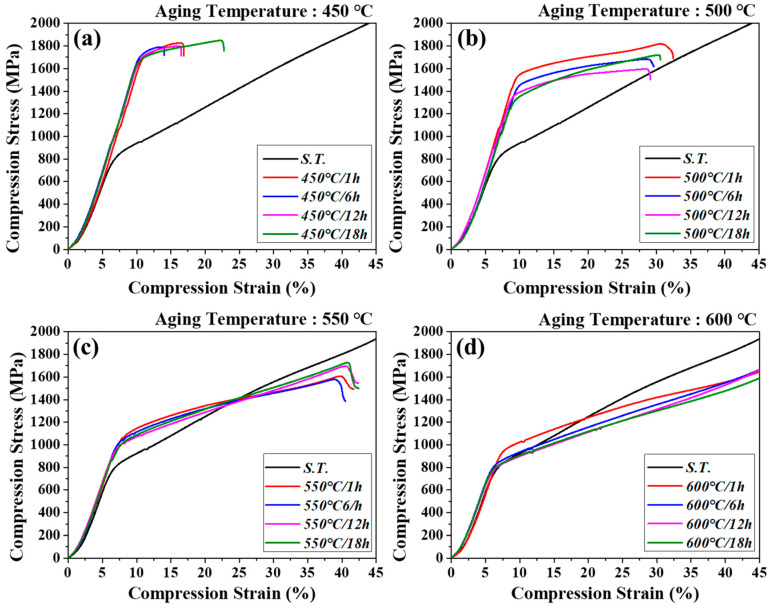
Compressive stress–strain curves of Ti-2Al-9.2Mo-2Fe alloy after solution treatment at 790 °C for 1 h and aging at (**a**) 450 °C, (**b**) 500 °C, (**c**) 550 °C, and (**d**) 600 °C for 1 h~18 h.

The results of room-temperature compression tests, conducted to analyze the effects of aging-induced precipitation phase changes on mechanical properties, are presented in Figure 11 and Table 3, along with the corresponding changes in compressive strength. The analysis focused on the aging temperature of 500 °C and aging time of 6 h, under which the hardness change was the largest. The 450 °C/6 h condition produced the largest yield strength (1690.0 MPa), indicating the occurrence of premature fracture. This was due to the influence of the isothermal ω phase precipitated during aging at lower temperatures, as mentioned earlier, which significantly increased the compressive yield strength but was accompanied by a decrease in the compressive elongation (13.1%), resulting in early brittle fracture. Subsequently, with the increase in aging time, the ω phase coarsened and transformed to the secondary α phase, decreasing the compressive yield strength (118.8 MPa) and increasing the compressive elongation (6.4%). However, the high ω phase fraction under the aging temperature of 450 °C produced insufficient precipitation driving force for phase transformation. Therefore, a high compressive yield strength (1571.2 MPa) and low compressive elongation (20.5%) were maintained even under the aging condition of 18 h.

For the aging condition of 500 °C, the isothermal ω phase was still precipitated and had a relatively high compressive yield strength. However, as the aging time increased, both the compressive yield strength and compressive elongation exhibited a decreasing trend. This was because the compressive yield strength decreased because of the coarsening and fractional reduction of the ω phase after aging for a long time, and the precipitated secondary α phase formed the grain boundary (GB) α phase. GB α has a very detrimental effect on crack initiation and propagation when its thickness exceeds microns [46]; therefore, as shown in Figure 8, the formation of a GB α phase under aging conditions of 1 to 12 h concentrated the stress between the α and β phase grain boundaries, which easily led to crack formation and propagation, significantly decreasing the compressive elongation [47,48]. However, for the 500 °C/18 h condition, the compressive elongation increased, as the WGB α phase formation due to the long aging time of 18 h dispersed the crack-propagation direction and reduced the crack-propagation speed. At higher aging temperatures of 550 and 600 °C, the compressive yield strength and compressive elongation were lower than or similar to that after solution treatment. This was because the WGB α phase formed around the grain boundaries grew and propagated significantly because of the high precipitation driving force, resulting in smoother crack propagation, and the basket-weave α phase precipitated within the grains also grew and propagated, resulting in very low compressive yield strength and high compressive elongation.

## 4. Conclusions

In this study, the precipitation phase was controlled through an aging process at various temperatures and times after the solution treatment of the Ti-2Al-9.2Mo-2Fe (2A2F) alloy, for application in transportation equipment parts such as automobile springs. The following results were obtained.

(1) The Ti-2Al-9.2Mo-2Fe alloy was solution-treated at 790 °C, which was below the β-transformation temperature, for 1 h and then rapidly cooled to form an equiaxed β-phase matrix and a fine primary α phase. The athermal ω phase was not precipitated.
(2) Aging treatment at temperatures of 450 and 500 °C resulted in precipitation of the isothermal ω phase, which decreased with increasing aging time. This showed that the ω/β phase boundary acted as a nucleation site between the aging temperature of 450 and 500 °C, causing a phase transformation from the ω phase to the α phase. In addition, for aging temperatures higher than 550 °C, the phase transformation was accelerated by the high precipitation driving force, so that the ω phase transformed into the secondary α phase within an aging time of 1 h, forming morphologies such as the WGB α phase and basket-weave α phase.
(3) It was confirmed that the precipitation and growth of the secondary α phase coarsened with the increase in aging temperature and aging time. The influence of aging temperature was more dominant, as evidenced by the comparison of the XRD and microstructure data, which exhibited a large difference of approximately 19.4% between the changes in the α-phase fraction according to the aging temperature (41.6%) and aging time (22.2%).
(4) The ω phase formed at aging temperatures of 450–500 °C improved the hardness and compressive strength, but lowered the ductility, causing brittle fracture. However, the secondary α phase decreased the hardness and compressive strength and increased the ductility, depending on the amount of precipitation and growth. In addition, when comparing the microstructures and compression test data of the 550 °C/6 h and 600 °C/6 h conditions, it was confirmed that the growth of the secondary α phase was more dominant in the mechanical properties.
(5) The Ti-2Al-9.2Mo-2Fe alloy, which is an LCB alloy, was able to exhibit excellent mechanical properties through the control of the precipitation phase by controlling the aging treatment. It was confirmed that the appropriate phase transformation from the ω phase to the secondary α phase occurred under the aging conditions of 500 °C/6 h and 500 °C/12 h, showing the best mechanical properties. This was thus deemed the optimal heat-treatment condition.

## Figures and Tables

**Table 1:** Chemical compositions of the Ti-2Al-9.2Mo-2Fe alloy (wt%).

Elements	Al	Mo	Fe	Cu	Si	C	H	O	N	Ti
Content (wt%)	2.33	9.33	1.98	0.015	0.037	0.0091	0.0013	0.054	0.0087	Bal.

**Table 2:** Phase fraction data of Ti-2Al-9.2Mo-2Fe alloy calculated using Equation (2) based on the XRD results.

	α Phase Fraction (%)	β Phase Fraction (%)	ω Phase Fraction (%)
450 °C/1 h	0.6	89.0	10.4
450 °C/6 h	0.8	87.9	11.3
450 °C/12 h	1.2	94.8	4.0
450 °C/18 h	1.2	96.1	2.7
500 °C/1 h	0.8	91.5	7.7
500 °C/6 h	20.3	76.1	3.6
500 °C/12 h	24.4	72.4	3.2
500 °C/18 h	31.8	67.6	0.6
550 °C/6 h	55.3	44.7	-
600 °C/6 h	45.6	54.4	-

**Table 3:** Compressive yield strength, ultimate compressive strength and compressive fracture strain of the Ti-2Al-9.2Mo-2Fe alloy.

	Compressive Yield Strength (MPa)	Ultimate Compressive Strength (MPa)	Compressive Fracture Strain (%)
450 °C/1 h	1615.3 ± 13.0	1825.5 ± 18.1	16.9 ± 2.1
450 °C/6 h	1690.0 ± 3.6	1874.7 ± 6.3	14.1 ± 0.7
450 °C/12 h	1583.8 ± 12.2	1777.2 ± 15.1	17.51 ± 0.8
450 °C/18 h	1571.2 ± 12.3	1723.0 ± 5.9	20.5 ± 1.4
500 °C/1 h	1458.7 ± 13.2	1786.7 ± 21.2	33.1 ± 1.8
500 °C/6 h	1375.9 ± 1.8	1680.7 ± 2.8	29.9 ± 0.1
500 °C/12 h	1314.7 ± 10.1	1633.1 ± 13.0	29.2 ± 0.0
500 °C/18 h	1242.6 ± 7.9	1710.7 ± 9.2	30.8 ± 0.2
550 °C/6 h	944.2 ± 17.6	1604.7 ± 24.4	40.3 ± 3.4
600 °C/6 h	746.8 ± 3.3	-	-

## Data Availability

The data presented in this study are available on request from the corresponding author. The data are not publicly available due to privacy and ethical restrictions.
